# Assessment of Intratumoral Doxorubicin Penetration after Mild Hyperthermia-Mediated Release from Thermosensitive Liposomes

**DOI:** 10.1155/2019/2645928

**Published:** 2019-03-07

**Authors:** Marc Derieppe, Jean-Michel Escoffre, Baudouin Denis de Senneville, Quincy van Houtum, Angelique Barten-van Rijbroek, Kim van der Wurff-Jacobs, Ludwig Dubois, Clemens Bos, Chrit Moonen

**Affiliations:** ^1^Imaging Division, University Medical Center Utrecht, Utrecht, Netherlands; ^2^Institut de Mathématiques de Bordeaux, UMR 5251, CNRS, Université de Bordeaux, Bordeaux, France; ^3^Maastro Lab, Maastricht University, Maastricht, Netherlands

## Abstract

In solid tumors, rapid local intravascular release of anticancer agents, e.g., doxorubicin (DOX), from thermosensitive liposomes (TSLs) can be an option to overcome poor extravasation of drug nanocarriers. The driving force of DOX penetration is the drug concentration gradient between the vascular compartment and the tumor interstitium. In this feasibility study, we used fibered confocal fluorescence microscopy (FCFM) to monitor in real-time DOX penetration in the interstitium of a subcutaneous tumor after its intravascular release from TSLs, Thermodox®. Cell uptake kinetics of the released DOX was quantified, along with an in-depth assessment of released-DOX penetration using an evolution model. A subcutaneous rat R1 rhabdomyosarcoma xenograft was used. The rodent was positioned in a setup including a water bath, and FCFM identification of functional vessels in the tumor tissue was applied based on AngioSense. The tumor-bearing leg was immersed in the 43°C water for preheating, and TSLs were injected intravenously. Real-time monitoring of intratumoral (i.t.) DOX penetration could be performed, and it showed the progressing DOX wave front via its native fluorescence, labeling successively all cell nuclei. Cell uptake rates (1/k) of 3 minutes were found (*n*=241  cells), and a released-DOX penetration in the range of 2500 *µ*m^2^·s^−1^ was found in the tumor extravascular space. This study also showed that not all vessels, identified as functional based on AngioSense, gave rise to local DOX penetration.

## 1. Introduction

Local drug delivery strategies in oncology aim at increasing delivery of anticancer drugs in the tumor, while limiting their exposure in healthy tissues that induces toxic side effects, e.g., cardiotoxicity of the small molecule doxorubicin (DOX, relative molecular mass of 544 Da) [1, 2]. One strategy devised in previous studies is the systemic injection of long-circulating nanomedicine formulations, such as pegylated liposomes, to facilitate drug accumulation in the tumor tissue [3, 4]. This can be achieved by exploiting two pathophysiological characteristics of solid tumors that constitute the enhanced permeability and retention effect (EPR) [5, 6]: (1) a higher vascular permeability because of a lack of vessel differentiation and (2) insufficient functional lymphatics. Despite this passive drug targeting, penetration of nanoparticles into the tumor interstitium is rather limited [7, 8] and variable according to the tumor type [3]. Moreover, nanoparticles display slow drug release in the tumor tissue, which often results in drug accumulation below the minimum concentration required to induce cell death [9].

The advent of temperature-sensitive liposomes (TSLs), with the first formulation described by Yatvin et al. [10], allowed the design of new drug delivery strategies with a local triggering by an external source of mild hyperthermia. These drug delivery systems must fulfill four main criteria: (1) a long plasma half-life, (2) a stable formulation at body temperature, (3) a phase transition temperature corresponding to a physiologically mild and safe hyperthermia, hence the name of low temperature-sensitive liposomes (LTSLs), and (4) a “fast” drug release rate from the liposomes when more than 50% of their payload was released within around half a minute when exposed to their phase transition temperature [11, 12]. Needham and coworkers were the first to incorporate lysolipids in the membrane bilayer and paved the way for an LTSL formulation that was clinically compatible [13].

Combination of LTSLs with an external heat source allowed local treatment, and Manzoor et al. introduced the concept of triggered, rapid intravascular release [14]: upon external triggering the LTSLs release the drug payload rapidly in the tumor vasculature and build up a drug concentration gradient between the tumor vasculature and the tumor tissue, which constitutes the driving force of diffusion that is the main source of transport for small molecules, e.g., DOX [15]. This drug penetration is proportional to the drug concentration gradient. Using TSLs with a fast drug release rate, one can therefore maximize drug penetration into the tumor.

To conduct drug distribution studies at the tissue scale, dorsal skin-fold window chambers have been used *in vivo*. These consist of a skin flap held vertically with an aluminium saddle that is sutured to the upper and the lower parts of the flap [16–18]. An optical window gives access to the fascial tissue and its vasculature, and tumor cells can be implanted into this window chamber. An alternative intravital imaging solution is fibered confocal fluorescence microscopy (FCFM); this modality allows real-time fluorescence imaging in a minimally invasive fashion by directly placing a fiber-based optical probe in contact with the tissue of interest in its natural physiological environment. FCFM is mainly used in clinical applications involving hollow organs, like in gastroenterology [19] and pulmonology [20], either visualizing the tissue based on its autofluorescence or after injection of fluorescein or indocyanine green.

In this study, we tested the feasibility to use FCFM to monitor *in vivo* in real-time DOX penetration in the tumor interstitium after intravascular release of DOX from the TSL (Thermodox®). The kinetic analysis from the time series allowed quantifying (1) the local uptake kinetics of released DOX in each individual cell of the interstitium after release from the TSL; (2) the kinetics of the apparent released-DOX penetration using the transport equation; and (3) the released-DOX deposition, the vascular washout, and the drug diffusion by means of an evolution model from the fluorescence signal intensity.

## 2. Materials and Methods

### 2.1. Experimental Setup

#### 2.1.1. Animals and Tumor Model

All procedures were performed according to the ethical guidelines and were approved by the animal welfare committee of Utrecht University (DEC 2014.III.03.035, Utrecht, the Netherlands). WAG/Rij rats were purchased from Charles River (Cologne, Germany). They were maintained at room temperature with 12 h light cycle in individually ventilated isolation cages and were fed ad libitum. The rats were 12 weeks old at the beginning of the experiments, weighing 250 g. Under gaseous anesthesia (Aerrane, Baxter, Deerfield, IL), a skin incision of a few millimeters was performed at the hind leg. Subsequently, rat R1 rhabdomyosarcoma tumor pieces (1–3 mm^3^) were subcutaneously implanted in the hind leg using a trocar. When the tumor volume reached 1500 *µ*m^3^, approximately after 3 weeks of tumor growth, the drug administration and imaging experiments were performed.

#### 2.1.2. Chemicals

Lysothermosensitive liposomal formulation of DOX (Thermodox®-TSL) at 2 mg/mL was obtained from Celsion Corp (Lawrenceville, NJ, USA). These nanoparticles release their payload as a burst in the temperature range of 39.5°C to 42°C, i.e., less than 5% of release at 37°C, and more than 65% at 41°C, within about 30 sec (*in vitro* data provided by Celsion Corp.). On the day of the real-time monitoring experiment, the Thermodox® solution was filtered using a PD10-desalting column (GE Healthcare Europe GmbH, Eindhoven, the Netherlands) to ensure that the DOX penetration that was monitored was fully encapsulated previously in the TSL. The rodents were administered intravenously with a Thermodox® dose of 4 mg/kg.

Doxorubicin hydrochloride (Sigma-Aldrich, St-Louis, MO) (relative molecular mass: 580 Da), named “free DOX” in this study, was injected intravenously at 4 mg/kg.

An intravascular fluorescence label, AngioSense 680 EX, was purchased from Perkin Elmer (Waltham, MA, USA). AngioSense is a 70 kDa near-infrared labeled-fluorescent polymer (excitation/emission wavelengths: 670/690 nm), which allows imaging the blood pool during the whole imaging session.

#### 2.1.3. Fibered Confocal Fluorescence Microscopy

**Figure 1:**
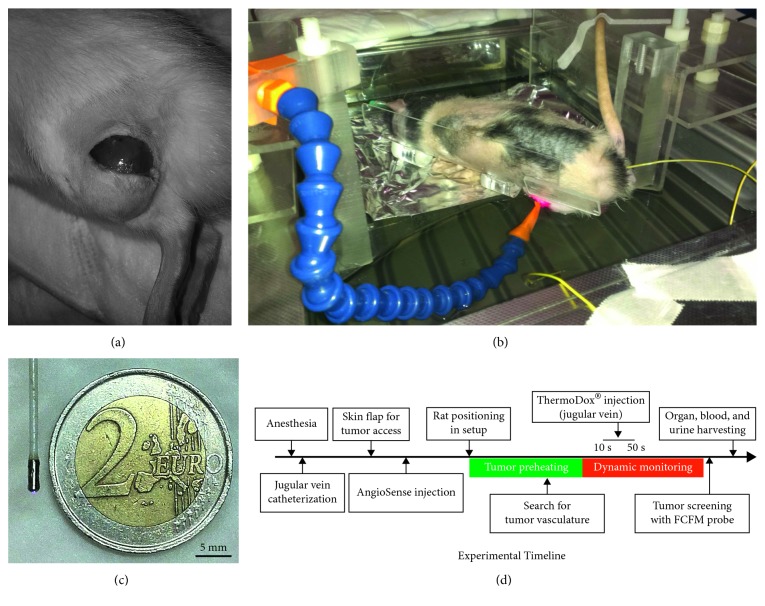
Tumor access (a), setup (b), tip of the FCFM probe (c), and timeline of the experiment (d). After incision of the skin at the tumor location (a), the rat was positioned on a platform placed at the water surface of the water bath, with the hind leg immersed in the water set to 43°C (b).

Fluorescence images were acquired in real-time (8.5 Hz) for 20 minutes using a dual-band FCFM system (Cellvizio® dual-band, Mauna Kea Technologies, Paris, France). Native fluorescence of DOX was collected with the 488 nm excitation channel, henceforth referred to as “green channel,” and blood vessels via AngioSense with the 660 nm channel, referred to as “red channel”. Their spectral sensitivity is 500–630 nm and 680–800 nm, respectively. A 1.5 mm diameter FCFM microprobe (PF-2210, Mauna Kea Technologies) was used (Figure 1(c)), with the following imaging specifications: lateral resolution 3.3 *μ*m, field of view (FOV) 602 *μ*m× 602 *μ*m, axial resolution 15 *μ*m, working distance 0 *μ*m. The tissue was excited at 4 mW of laser power for both channels.

#### 2.1.4. Animal Positioning

A water bath (Memmert, Schwabach, Germany) was used to ensure a mild and homogeneous tumor heating (Figure 1(b)). The water temperature was monitored using fiber optic temperature sensors (Luxtron, Lumasense Technologies GmbH, Frankfurt, Germany). A custom-made platform was designed to position the animal above the water surface and to immerse only the tumor bearing leg (Figure 1(b)).

The animal was thermally isolated from the water bath using a piece of aluminium foil (Figure 1(b)). The rectal temperature and the water temperature (set to 43°C) in the vicinity of the tumor were monitored during the session. During the whole experiment, the rectal temperature was lower than the phase transition temperature of the TSL (Figure S1). The hind leg bearing the tumor was positioned in the water bath for 10 minutes, which was sufficient to get the tumor tissue at 43°C (data not shown).

Handling of the FCFM microprobe was facilitated by using a modular hose, holding it in fixed position in contact with the tumor tissue, such that a dynamic microscopy time series of a fixed tumor location could be obtained (Figure 1(b)).

#### 2.1.5. Timeline of the Imaging Session

The rats were anaesthetized with an i.p. injection of 75 mg/kg of ketamine (Narketan, Vetoquinol, 's-Hertogenbosch, the Netherlands) and 0.25 mg/kg of dexmedetomidine (Dexdormitor, Orion Pharma, Mechelen, Belgium). Then, the jugular vein was catheterized, a 1 cm skin flap was created at the tumor level (Figure 1(a)), and the rats were then injected intravenously with 192 nmol·kg^−1^ of AngioSense (Figure 1(d)).

Subsequently, the rats were positioned on the platform of the water bath, laying on the flank, with the tumor bearing leg immersed in the 43°C water. Temperature probes were then placed to monitor the temperature of the water bath and the body temperature of the animal. The tip of the FCFM probe was placed manually to make contact with the tumor tissue in the water, and the tumor was explored until tortuous tumor vessels were found. Then, stability of the FOV was verified by waiting one minute and by checking any motion of the tumor microvasculature in the image. The syringe containing Thermodox® was only then placed in the catheter, to avoid premature heating of the liposomes. At this point, the 20-minute real-time monitoring was started. After collecting the 10-second baseline with tissue autofluorescence, a 4 mg/kg bolus injection of TSLs, or free DOX, was administered intravenously in the jugular vein in around 40 s.

After completion of the dynamic sequence, exploration of the tumor surface was performed manually with the FCFM probe, and micrographs were acquired to evaluate the presence of DOX in the tumor microenvironment by means of its native fluorescence [21, 22].

At the end of the session, the rats were sacrificed by an i.p. injection of 200 mg/kg of pentobarbital (Euthanimal 20%, Alfasan, Woerden, the Netherlands), and the blood, the urine if any, and the tumor were harvested.

Two treatment groups consisted of the “free DOX” group (*n*=3) and the “TSL” group (*n*=5).

## 3. Histopathology and Liquid Biopsy Analysis

Tumors were harvested and fixed in the formol-acetic acid solution. Then, histological samples were embedded in paraffin, cut at approximately 5 *μ*m, and prepared using conventional hematoxylin/eosin protocol. The tissue sections were examined by light microscopy on a Keyence microscope (Keyence International, Belgium). Whole tumor imaging was performed using a 4x magnification objective (numerical aperture 0.6) and the mosaicking module with a 7-by-7 matrix. The size of the neoplastic nodule on the slide was measured with a ruler. Then, micrographs of each tumor area (necrotic versus proliferative areas) were acquired at 20x magnification using a PlanFluor objective (Numerical aperture 0.5) and at 60x using an oil-immersion PlanApo VC objective (numerical aperture 1.4).

Micrographs of DOX fluorescence in frozen-tissue sections were acquired using a Leica TCS SP8 X confocal fluorescence microscope, with a 10x magnification objective, a 504 nm excitation wavelength, and an emission filter of 540–680 nm.

DOX concentration in blood and urine samples was measured using Ultra Performance Liquid Chromatography (UPLC) with fluorescence and UV detection. An ACQUITY UPLC BEH C18 separation column was used (130 Ångström, size of 1.7 micron, 1.7 × 50 mm). The mobile phase consisted of an eluant with the following mixture: 75% reverse-osmosis water, 25% acetonitrile, and 1% perchloric acid. During separation, a column temperature of 50°C and a sample temperature of 25°C were set, with 3 minutes of run time. The fluorescence was measured with a 480 nm excitation/565 nm emission and a UV detection of 234 nm.

## 4. Kinetic Analysis of Released-DOX Penetration

The real-time fluorescence image data obtained was processed offline using MATLAB® 2013 (The MathWorks, Natick, MA, USA). To increase the signal-to-noise ratio, the sequence was averaged temporally to an 8 s frame rate. Kinetic analysis of DOX penetration consisted of the 3 following independent but complementary sections.

### 4.1. Cell Uptake Kinetics of Released-DOX

Cell uptake kinetics was assessed using the dedicated parametric pipeline described in Derieppe et al. [23]. Briefly, this automated pipeline includes cell detection, which was facilitated by a nonlocal means algorithm [24], a cell tracking with the iterative-closest-point algorithm [25], and a fitting of the resulting uptake profiles by means of a two-compartment model where the fluorescence signal intensity (*I*) is as follows [26]:

(1) $$\begin{array}{c} I\left(t\right)=A\left[1-e^{-k\left(t-T\right)}\right], \end{array}$$

where *A* is the maximum DOX fluorescence signal, *T* the time of signal onset, and *k* the uptake rate. The model was considered accurate when Pearson's correlation coefficient (*r* ^2^) was greater than 0.95; the values of the uptake rate of released DOX were reported as median with interquartile ranges. Then, a statistical analysis of the resulting pharmacokinetic parameters of the cell population visible in the FOV was performed.

### 4.2. Analysis of the Released-DOX Penetration in the Tumor Interstitium

Quantitative evaluation of released-DOX penetration in the tumor interstitium was then performed by estimating the instantaneous apparent DOX transport (noted $\vec{V}$) using the transport model applied to all acquired images [27]. In practice, the following equation was applied in a homogeneous environment to calculate the instantaneous released-DOX penetration between timepoints *t* and *t *+* δt*:

(2) $$\begin{array}{c} I_{t}+\vec{V}\cdot \vec{\nabla }I=0, \end{array}$$

where *I* denotes the native fluorescence signal monitored in the green channel and *I*_*t*_ the partial temporal derivative of *I* calculated between the timepoints *t* and *t *+* δt*. The left part of this equation consists of a transient term (*I*_*t*_) and an apparent transport $\vec{V}\cdot \vec{\nabla }I$, which stand for any temporal and spatial grey intensity variations, respectively. The sum of both terms equal 0 to ensure the signal intensity conservation with motion in the field of view. Any spatiotemporal intensity variations occurring between timepoints *t* and *t *+ *δt* may be attributed to DOX transport in the model used. The estimated transport field $\vec{V}$ thus accounts for spatiotemporal fluorescence intensity variation occurring during the dynamic imaging sequence.

The transport model of equation (2) is intrinsically underdetermined, thus leading to an ill-conditioned numerical scheme. The transport field $\vec{V}$ was therefore computed on a pixel-by-pixel basis by applying the minimization process as follows:

(3) $$\begin{array}{c} \arg\underset{\vec{V}}{\min}\int_{Ω}\left|I_{t}+\vec{V}\cdot \vec{\nabla }I\right|+\alpha \left({\left\|\vec{\nabla }u\right\|}_{2}^{2}+{\left\|\vec{\nabla }v\right\|}_{2}^{2}\right)\;d\vec{r}, \end{array}$$

where Ω ⊆ *ℝ* ^2^ is the coordinate domain of the image, (*u*, *v*) the components of transport vectors estimated, and $\vec{r}$ ∈ Ω the spatial location. The minimized functional consists of both additive contributions as follows: (1) a fidelity term (left part of the integral in equation (3)) that optimizes, through an *L* ^1^ norm, the transport model of equation (2); given that an *L* ^1^ penalizer is applied, transient variations occur identically, and regardless of the grey level intensity, (2) a regularization term (equation (3), right part of the integral) designed to introduce a sufficient conditioning to the numerical scheme. The regularization term is given by ${\left\|\vec{\nabla }u\right\|}_{2}^{2}=u_{x}^{2}+u_{y}^{2}$ and ${\left\|\vec{\nabla }v\right\|}_{2}^{2}=v_{x}^{2}+v_{y}^{2}$, *u*_*x*_, *u*_*y*_, *v*_*x*_ with *v*_*y*_ being the spatial partial derivatives of *u* and *v*, respectively. Physically, this regularization term assumes that the transport between neighboring pixels is moderate.

The data fidelity and the regularization terms are linked by the weighting factor *α* that was set to 1.0 in the scope of this study. This value was motivated by the fact that a high α value increases the stability of the numerical scheme but also hampers in turns the “spatial elasticity” of the estimated displacement field. In order to optimize the computation time and to ensure the convergence of the algorithm, a multiresolution scheme that iterated the registration algorithm was used from a four-fold down-sampled image step-by-step to the full image resolution [28]. The interested reader is referred to Corpetti et al. [29] for more information about the numerical resolution of dense estimation of fluid flows.

In order to mitigate the local impact of the nucleus fluorescence signal on DOX penetration in the interstitium, a spatial low-pass Butterworth filter was applied to each individual image before the resolution of equation (3). In order to remove cell nuclei with typical diameters up to 10 *μm*, according to an image pixel size of 1.0 × 1.0 *µ*m^2^, the cut-off frequency *f*_c_ of the low-pass filter was set to *f*_c_=*f*_0_/16, *f*_0_ being the original image sampling frequency.

The onset of fluorescence signal was defined when the maximum fluorescence signal in the current image exceeded at least 5% of the maximum signal of the sequence. Since the transport model of equation (2) relies on the spatiotemporal fluorescence intensity, the analysis of the released-DOX penetration in the interstitium was performed on images acquired after this timepoint.

A principal component analysis (PCA) was applied subsequently on the time series of the computed 2-dimension penetration vector fields in order to find the spatial orthogonal basis; the resulting principal axis served to generate an adequate representation of the sequence of displacement fields. An averaged motion amplitude along each principal axis was then calculated for each individual displacement field and allowed calculating the temporal profile of the average motion amplitude along each principal axis.

### 4.3. Modeling of Released-DOX Penetration

Ultimately, the spatiotemporal distribution of released-DOX fluorescence signal intensity *S* was modeled and included a uniform released-DOX deposition *δ*, a vascular washout *ω* proportional to the current fluorescence signal intensity, and a homogeneous released-DOX apparent diffusion *ν* in the FOV, with the following equation:

(4) $$\begin{array}{c} \frac{\partial }{\partial t}S\left(\vec{r},t\right)=\delta -\omega \cdot V\left(\vec{r}\right)\cdot S\left(\vec{r},t\right)+\nu \cdot \Delta S\left(\vec{r},t\right), \end{array}$$

where Δ is the Laplacian operator, *t* is the time instant, $\vec{r}$ = (*x*, *y*, *z*) is the voxel coordinate, and $V\left(\vec{r}\right)$ is a probability density function (PDF) that has a value close to 1 (0, respectively) in voxels having a high (low, respectively) probability to be in the vascular space. Here, only diffusion was considered, as diffusion is dominant for small molecules, such as doxorubicin, in the extravascular space [15].

In this model, the fluorescence signal intensity $S\left(\vec{r},t\right)$ was collected in the green channel. The PDF $V\left(\vec{r}\right)$ was derived from the fluorescence signal in the vessels collected in the red channel at the beginning of the acquisition. This signal was normalized between 0 and 1 and assumed to be temporally invariant (a linear relation between the fluorescence signal in the vessels and the PDF was assumed to be a good approximation in the scope of this study). The coefficients *δ*, *ω*, and *ν* were assumed to be spatially and temporally invariant within a temporal window covering the time period starting from 100 seconds, which corresponds to the onset of fluorescence signal in the FOV, to 20 minutes of imaging.

Equation (4) was solved using a finite difference method integrated in an explicit Euler scheme in order to simulate the evolution of fluorescence signal intensity for specific values of *δ*, *ω*, and *ν*. Using a Levenberg–Marquardt fit, the coefficients *δ*, *ω*, and *ν* were calculated to minimize the least-square residue between the model and the measured data over the complete sequence. The algorithm applied multiple regularly sampled initial conditions for *ν* in order to prevent the algorithm from falling into local minima. The goodness of the fit, as evaluated by Pearson's correlation coefficient, was computed in order to evaluate whether the model described accurately the fluorescence signal enhancement.

Since cell nuclei are nonmoving structures, the impact of their fluorescence signal was reduced using a spatial low-pass Butterworth filter (order 1) in each individual image before the numerical resolution of equation (4). Similar to section 4.2, the cut-off frequency *f*_c_ of the low-pass filter was set to *f*_c_=*f*_0_/16.

## 5. Results

### 5.1. Histopathological Analysis

To evaluate the influence of mild hyperthermia on tumor tissue, histopathological analyses were carried out at the end of our imaging session. Tumors were visible as rounded neoplastic nodules present within the subcutaneous and muscle tissues and partially surrounded by thin fibrous capsules. All tumors (37°C vs 43°C) exhibited common histopathological characteristics. The proliferation areas were made up of poorly differentiated neoplastic cells within a fine vascular stroma. These cells were highly pleomorphic with one or more prominent nuclei. Some multinucleated cells were also observed in all tumors. The transition proliferative/necrotic areas are well-defined, with a loss in cell density and a clear fibrillary aspect that is characteristic of the extracellular matrix in the necrotic area (25–30% of all tumor nodules). Few multifocal infiltrations of neutrophils were observed in all tumors. No apparent tissue damage in any of the areas exposed to a 43°C local hyperthermia (Figure S2) was observed in comparison to the control tumors (Figure S2).

### 5.2. Real-Time Monitoring of Released-DOX Penetration

**Figure 2:**
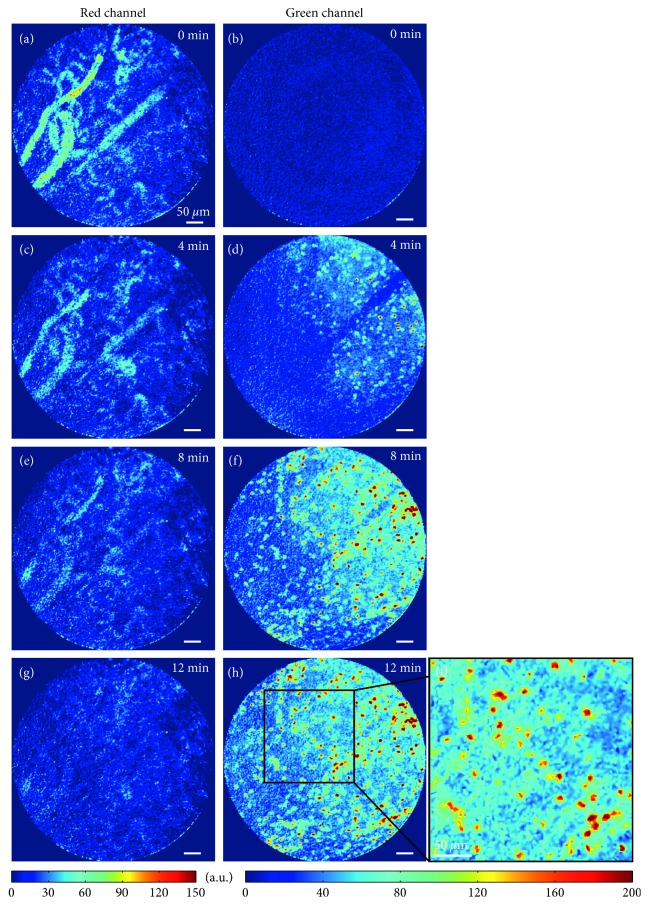
Real-time monitoring of doxorubicin penetration in the tissue microenvironment after TSL intravascular release (a, c, e, g). Red channel with AngioSense^TM^ (blood-pool labeling) showed that the acquisition was performed in a steady FOV and that the vessels were functional (b, d, f, h, i). The green channel shows DOX fluorescence signal enhancement after the bolus injection of TSL.

In the red channel, staining of the blood pool by AngioSense allowed finding functional, characteristically tortuous, tumor microvasculature (Figures 2(a), 2(c), 2(e), and 2(g)). In the free DOX group, i.v. injection did not lead to a detectable FCFM signal (*n*=3), neither during the real-time monitoring (Figure S3) nor during histopathology (Table S1). Conversely, in the TSL group (*n*=5), a fluorescence signal enhancement was collected (*n*=2, Table S1), but not in the other animals (*n*=3). Due to image drift in one animal, only the onset of DOX fluorescence increase could be analyzed but not the complete DOX dynamics and distribution.

The fluorescence signal enhancement was present in the extravascular space, as well as in the cell nuclei of the tumor interstitium, which reflected intracellular uptake of released DOX. Interestingly, the onset of DOX uptake in the cells started from one side and progressively spread to involve cells throughout the FOV (Figures 2(b), 2(d), 2(f), 2(h), and 2(i)). However, no DOX fluorescence enhancement was visible in vessels. In the red channel, the fluorescence of blood vessels decreased during the acquisition probably owing to photobleaching, but it was still possible to locate them until 12 minutes of acquisition, indicating that the position of the FOV was steady.

### 5.3. Cell-Uptake Kinetics of Released-DOX

**Figure 3:**
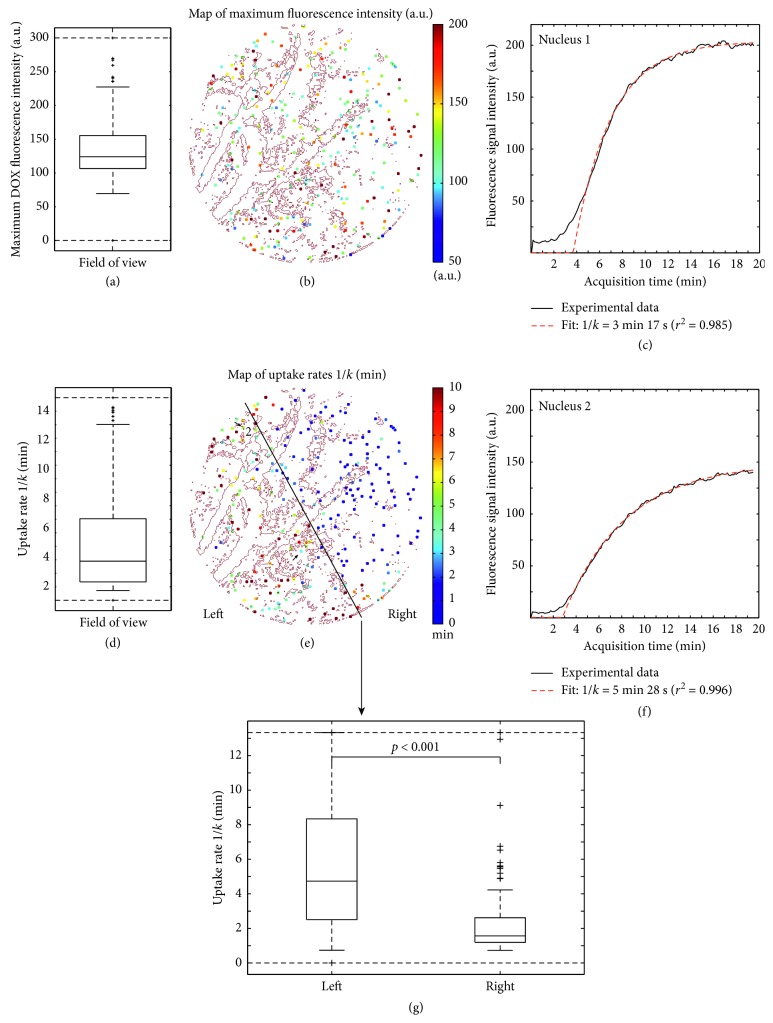
Uptake rates 1/*k* derived from the two-compartment model. Boxplots of the maximum DOX fluorescence intensity (a) and the uptake rate 1/*k* (d) and their corresponding spatial distributions, respectively (b, e). Characteristic examples of fluorescence signal enhancements collected in two nuclei (dark plain curves) and the fitted curves derived from the two-compartment model (dashed red curves) (c, f). Two distinct subpopulations of uptake rates in the FOV (e) are significantly different (g).

Real-time monitoring of released-DOX penetration in the tumor interstitium shows cell nuclei that display increasing fluorescence signal (Figures 2(b), 2(d), 2(f), 2(h), and 2(i)), thus indicating an increasing DOX concentration upon cell uptake. 241 cell nuclei could be detected and were included in the analysis. The maximum fluorescence intensity derived from the fit (124 a.u., with interquartile range of 49 a.u.) (Figure 3(a)) did not display any spatial correlations in the maximum-fluorescence-intensity map, neither in the FOV, nor with the position of the vessels in the FOV (Figure 3(b)). Of all nuclei, the median of uptake rates 1/k was 2 minutes 54 s (interquartile range 4 minutes 40 s) (Figure 3(d)); however, remarkably faster uptakes (range 0–3 minutes 20 s) were observed in the upper-right half of the FOV (Figure 3(e)). In this case, no clear spatial correlation with the microvasculature could be identified (Figure 3(e), red dotted lines in background), indicating that released-DOX inducing the fluorescence signal did not originate from the microvessels visible in the FOV, but instead from vessels beyond the confocal slice.

### 5.4. Analysis of the Interstitial Released-DOX Penetration

**Figure 4:**
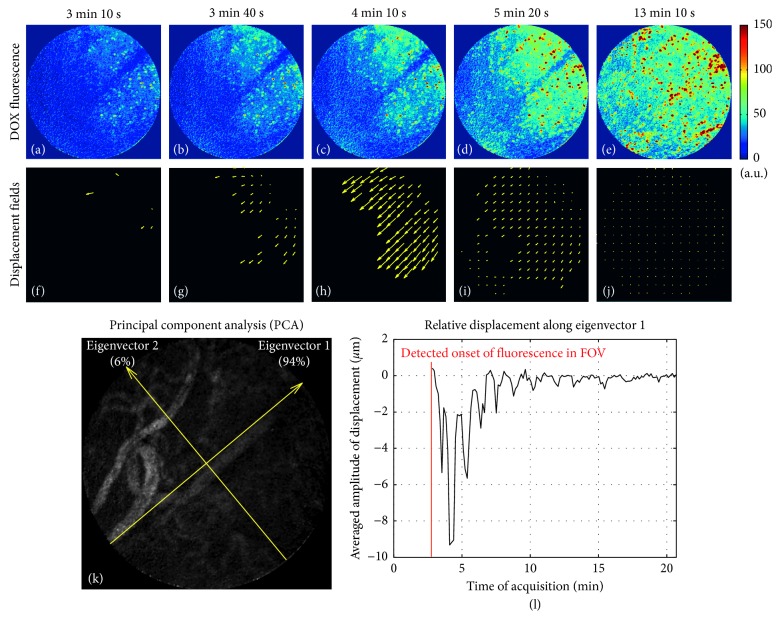
Assessment of released-DOX penetration derived from the implemented fluid dynamics model. (a–e) Fluorescence images measured at 3 min 10 s (a), 3 min 40 s (b), 4 min 10 s (c), 5 min 20 s (d), and 13 min 10 s (e). (f–j) Corresponding released-DOX penetration shown by the displacement field. For an easier visualization, only displacement vectors associated to voxels with sufficient fluorescence signal (i.e., greater than 5% of the maximum fluorescence signal intensity) are displayed. The principal component analysis allowed determining the main direction of released-DOX penetration, with 94% of relative displacement along the eigenvector 1 (k). The temporal profile of relative displacements along the eigenvector 1 equilibrated 7 minutes after the fluorescence onset (l).

The spatiotemporal distribution of the released-DOX native fluorescence directly reflected released-DOX penetration (Figures 4(a)–4(e)) and was assessed using the fluid dynamics model described by equation (2) through the minimization process of equation (3). The arrival of DOX was visible in the upper-right corner of the FOV 3 minutes after bolus injection and pointed toward the lower-left corner of the FOV (Figure 4(i)).

The principal component analysis allowed determining the main direction of released-DOX penetration, with 94% of relative displacement along the principal axis 1 (Figure 4(k)). The temporal profile of relative displacement along the principal axis 1 displayed a periodic pattern (Figure 4(l)). Released-DOX penetration then equilibrated in the FOV 4 minutes after the detected fluorescence onset, according to the low-amplitude displacement vectors (Figure 4(j)).

### 5.5. Modeling of Released-DOX Penetration

**Figure 5:**
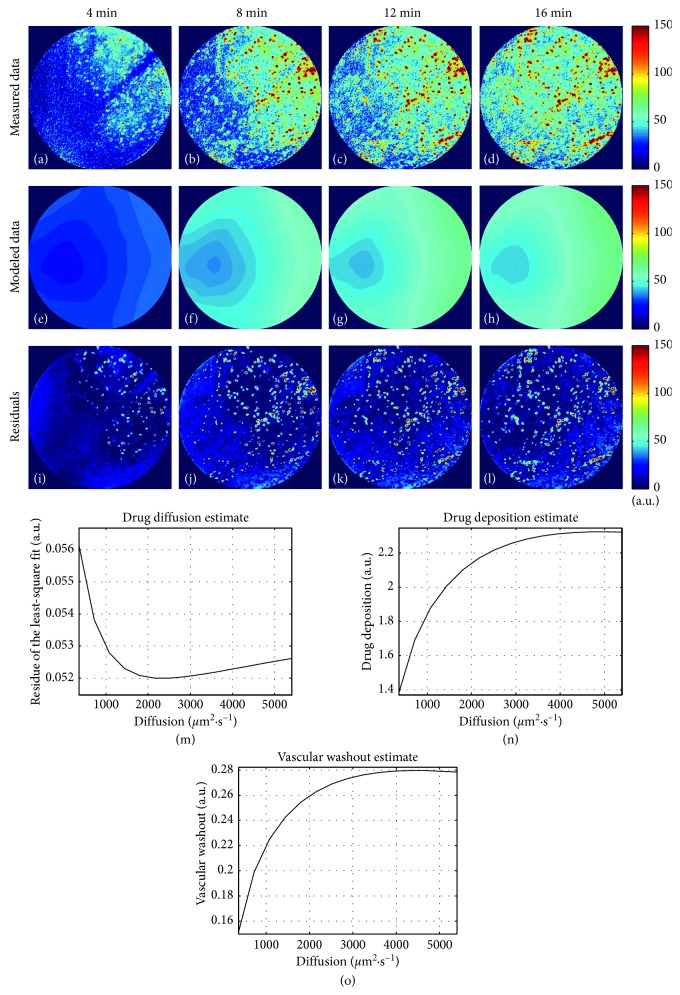
Modeling of released-DOX penetration in the tumor microenvironment. The implemented evolution model allowed calculating a drug diffusion of *ν* = 2500 *µ*m^2^·s^−1^ (e–h). Only the background fluorescence served in the evolution model, as shown by the absolute difference maps (i–l) calculated between the experimental data and the computed released-DOX penetration by the evolution model. The parameters to apply were calculated for the released-DOX penetration model (i.e., equation (4)). The *x*-axis reports regularly sampled values of the diffusion parameter *ν* that are exhaustively enumerated during the optimization process. (m) The optimization of the released-DOX apparent diffusion parameter *ν*: the averaged least-square residue between the model and the measured data was minimal for *ν* = 2500 *µ*m^2^·s^−1^. Optimal released-DOX deposition *δ* and vascular washout *ω* are displayed for each value of *ν* in (n) and (o), respectively.

Released-DOX penetration was modeled using the evolution model of equation (4) (Figure 5). The quality of the fit, as computed by the sample Pearson's correlation coefficient, was equal to 0.89, reflecting a close match of our proposed model with the experimental data. It is to be noticed that this model only used the background fluorescence as input, with a negligible impact of the nucleus fluorescence observed in the acquired sequence (Figures 5(a)–5(d)); this was confirmed by the maps of the residuals (Figures 5(i)–5(l)), i.e., the difference between the experimental data and the evolution model. The estimated drug diffusion *ν* was approximately 2500 *µ*m^2^·s^−1^ (see Figure 5(m)).

### 5.6. Observation of the Tumor Surface by FCFM

After monitoring dynamically DOX penetration after intravascular release at a single location, manual exploration of the tumor surface by the FCFM probe showed heterogeneity of DOX distribution in the tumor (Table S1). Interestingly, the native fluorescence of DOX was predominantly located close to the vessels imaged in the red channel (Figure S4a) and showed a pattern similar to that found during the real-time monitoring with high signal from the cell nuclei (Figure S4b). However, despite the presence of tortuous vessels (Figure S5a), a nonnegligible amount of locations in the same tumor did not show any DOX fluorescence (Figure S5b), suggesting heterogeneity in DOX distribution in the tumor. In these cases, after sacrifice of the animal, a tumor incision allowed insertion of the FCFM probe for screening of the tumor tissue and also showed high DOX heterogeneity within the tumor tissue (data not shown); this heterogeneity was confirmed in DOX fluorescence micrographs collected from ex vivo samples (Figure S6).

## 6. Discussion

Evaluation of drug penetration in the tumor microenvironment is key to optimize therapeutic strategies of hyperthermia-triggered drug delivery in solid tumors. In this feasibility study, a setup was devised to monitor DOX penetration in the tumor interstitium in real time after intravascular release of DOX from TSL. Here, we used FCFM as an alternative of commonly used dorsal skinfold window chambers to assess drug penetration in a mechanically unrestricted tumor microenvironment.

First, no apparent microstructural changes could be observed in tumor tissue exposed to a 43°C mild hyperthermia, thus confirming the noninvasive nature of the hyperthermia procedure. Real-time imaging using FCFM was performed at the tumor surface with a skin incision and allowed imaging separately the vascular compartment and released DOX after intravascular release from the TSL.

In the TSL group, the real-time monitoring of DOX was successful in 2 out of 5 cases in the TSL group only, but DOX was found in every tumor when exploring the tumor rim at the end of the imaging session. In the free DOX group, no DOX could be detected, neither during the real-time monitoring nor during the tumor exploration at the end of the imaging session, suggesting that the DOX concentration was too low to be detected in the extravascular space. This could be explained by a low concentration gradient between the tumor vasculature and the extravascular space, thus limiting free DOX penetration, hence the use of TSLs.

Released-DOX penetration kinetics could be assessed upon imaging of cell-uptake kinetics of released DOX in the tumor interstitium and DOX diffusion coefficient, both in the same dataset. Here, 241 cell nuclei could be detected using the image processing pipeline developed previously [23], thus allowing a statistical analysis. Uptake time constants of 3 minutes were found after injection of 4 mg/kg DOX encapsulated in TSLs. Interestingly, no apparent link could be made between the location of the microvasculature and the direction of the propagation front when observing cell uptake of DOX in the FOV. The uptake rates around 3 minutes show faster uptakes than those found in previous studies where uptake rates 1/k around 10 minutes were found with free DOX [30, 31]. This would confirm the usefulness of TSLs, where a TSL intravascular release induces higher DOX levels in the extravascular space and thus higher DOX concentration gradients as a driving force for the faster cell uptake.

Using the transport model of equation (2) together with a principal component analysis, the released-DOX penetration in the presented results was shown to occur mainly along one axis oriented from the top-right to the bottom-left corner of the FOV (Figure 4(k)). Relative DOX displacement was found to equilibrate 7 minutes after the detected DOX arrival in the FOV. This finding would mean that the concentration gradient, which is the driving force for DOX penetration into the tumor interstitium, was not present anymore between the vascular compartment and the interstitium after 7 minutes. The estimated relative displacements here indicate that like in the cell uptake kinetics of released-DOX computed above, no spatial correlation between the displacement field and the location of the microvasculature was found. In the scope of this study, it is important to underline that an apparent displacement is calculated here, without taking the fluid rheology into account. This will be worth considering in future studies.

It is important to underline that any spatiotemporal intensity variations occurring between timepoints *t* and *t *+* δt* in equation (2) may be attributed in our model to “displacement”. This assumption brings a limitation with regards to released-DOX penetration in the interstitium, which is likely to be biased by the fluorescence signal in the cell nuclei, as these are nonmoving image structures. Our simulations were thus conducted on spatially filtered fluorescence images in order to circumvent this issue. Therefore, it is to be noted that the apparent DOX velocity estimates logically increased when a reduced cut-off frequency *f*_c_ was applied in the low-pass spatial-image filtering process because, in this case, nuclei weighted less in filtered images. Using *f*_c_=*f*_0_/16 and *f*_c_=*f*_0_/8, DOX velocity estimates displayed less than 10% of variations, thus confirming that the local impact of the fluorescence signal in the cell nuclei was properly discarded.

The diffusion coefficient reflects the magnitude of driving force generated by the concentration gradient built up between the vascular compartment and the tumor interstitium. The model of released-DOX penetration yielded a diffusion estimate around 2500 *µ*m^2^·s^−1^. In the present study, another source of uncertainty arose from the linear intensity of the vessel likelihood that was assumed to be located along the red fluorescence signal intensity (equation (4)). Consequently, a small but nonnegligible drug evacuation is also assumed in the interstitium, where positive nonzero values are found in the images in the red channel. It is important to report that, for instance, a greater diffusion coefficient would logically be found if no evacuation in the interstitium is assumed in our model. Nonetheless, this diffusion estimate is greater than the 40 *µ*m^2^·s^−1^ reported in previous studies using free DOX [9, 30], or 150 *µ*m^2^·s^−1^ found by Swabb et al., who established the correlation between the tissue diffusion coefficient at 37°C and the solute molecular weight, i.e., 544 Da for DOX [32]. This one-order-of-magnitude discrepancy may be in line with the use of TSLs because the payload released locally yields higher DOX concentration in the vasculature, thus increasing DOX concentration gradient between the vasculature and the extravascular space, and in turn a higher diffusivity.

Another source of bias in the evaluation of the diffusion coefficients may here arise from the 2D observation of a 3D diffusion process; this 2D observation is due to the design of the FCFM. Hence, only a 2D implementation of equation (4) could be applied, that neglects out-of-plane drug diffusion. Given that the FCFM probe used here allowed imaging from 0 to 15 *μ*m of depth, this bias could only come from out-of-plane events occurring deeper than the 15 *μ*m of confocal slice.

After monitoring dynamically DOX penetration at a single location, the manual exploration of the tumor surface and its depth by the FCFM probe showed a clear spatial heterogeneity in DOX native fluorescence, despite the presence of tortuous vessels and the homogeneous heating ensured by the water bath.

Despite the detection of functional vessels with AngioSense, no real-time DOX penetration could be observed by FCFM in 3 out of the 5 animals in the TSL group whereas histopathology showed high DOX concentration in the tumor area in all animals (*n*=5, Table S1). In addition, as clearly shown in Figure 2, DOX penetration does not arise from all functional vessels. Vessel functionality is identified in this study as showing AngioSense fluorescence. It should be noted that AngioSense has been injected well before the TSL injection and that its blood half-time is long (around 7h, data of supplier). Taken together, these data indicate that vessels with AngioSense-based functionality are not all functional with respect to DOX penetration upon TSL injection. Two possible mechanisms could explain this: either TSL did not (yet) arrive in the FCFM observed region or the TSL already lost their DOX payload upstream before arrival. The latter explanation may be relevant because of the very fast release of DOX from TSL upon hyperthermia (on the order of seconds) and should be considered when combining hyperthermia and TSLs for regional drug delivery.

Our FCFM and data processing approach could also serve for the study of extravasation of drug nanocarriers, e.g., its enhancement by hyperthermia [33], provided that the nanocarriers can be sufficiently fluorescently labeled. In this study, however, we followed the tumor preheating protocol in order to maximize drug uptake in the tumor, which leads to immediate intravascular release from the liposomes [34]. It is noteworthy that this approach may lead to a better understanding of the relation between drug penetration and tumor physiology characteristics, such as the interstitial fluid pressure [35] and the solid stress described recently [36]. Last, the image processing framework conducted in this study can be used in a broad range of experiments where imaging modalities at the tissue scale allow collecting pharmacokinetic parameters for the evaluation of drug formulations and drug nanocarriers [37].

## 7. Conclusions

FCFM can provide real-time visualization of DOX *in vivo* in a mechanically unrestricted tumor microenvironment, allowing the study of DOX penetration at the tissue scale. The proposed image analysis framework provides data on cell uptake kinetics as well as penetration kinetics of released DOX and thus helped to characterize the driving force constituted by the concentration gradient after intravascular release of DOX from the TSL.

## Data Availability

The data used to support the findings of this study are available from the corresponding author upon request.

## Supplementary Materials

Supplementary Materials Table S1: summary of the results rat-by-rat. DOX concentrations in urine and blood correspond to the results of single quantitative measurements. Figure S1: representative profiles of rat rectal temperature recorded during real-time monitoring. Figure S2: micrographs showing the impact of mild hyperthermia on the tumor microstructure in the proliferative area (b, c, i, j), the transition area (d, e, k, l), and the necrotic area (f, g, m, n) of the rat 4. No morphological damage was observed at 43°C (h–n) compared to 37 C (a–g) in any of the 3 areas. Figure S3: real-time monitoring of doxorubicin penetration in the tissue microenvironment after free DOX intravenous injection (a, c, e, g). Red channel with AngioSenseTM (blood-pool labeling) showed that the acquisition was performed in a steady FOV (b, d, f, h). No DOX fluorescence signal enhancement could be observed in the green channel after free DOX intravenous injection. Figure S4: micrographs collected after dynamic monitoring. In the interstitium, nuclei that take up doxorubicin (b) are predominantly located around the vessels (a). Figure S5: micrographs acquired after dynamic monitoring. Despite the presence of tortuous vessels characteristic of tumor tissue (a), a nonnegligible amount of areas in the tumor tissue did not show any doxorubicin signal (b). Figure S6: representative fluorescence micrographs (mosaicking) of a tumor tissue exposed to TSL. DOX distribution is heterogeneous. Objective: 10x.
