# Supplementary material for: Assessment of Intratumoral Doxorubicin Penetration after Mild Hyperthermia-Mediated Release from Thermosensitive Liposomes
Source: Contrast Media Mol Imaging. 2019 Mar 7;2019:2645928. doi: 10.1155/2019/2645928 (PMC6431439; doi:10.1155/2019/2645928)
Supplement: Supplementary Materials — Table S1: summary of the results rat-by-rat. DOX concentrations in urine and blood correspond to the results of single quantitative measurements. Figure S1: representative profiles of rat rectal temperature recorded during real-time monitoring. Figure S2: micrographs showing the impact of mild hyperthermia on the tumor microstructure in the proliferative area (b, c, i, j), the transition area (d, e, k, l), and the necrotic area (f, g, m, n) of the rat 4. No morphological damage was observed at 43°C (h–n) compared to 37 C (a–g) in any of the 3 areas. Figure S3: real-time monitoring of doxorubicin penetration in the tissue microenvironment after free DOX intravenous injection (a, c, e, g). Red channel with AngioSenseTM (blood-pool labeling) showed that the acquisition was performed in a steady FOV (b, d, f, h). No DOX fluorescence signal enhancement could be observed in the green channel after free DOX intravenous injection. Figure S4: micrographs collected after dynamic monitoring. In the interstitium, nuclei that take up doxorubicin (b) are predominantly located around the vessels (a). Figure S5: micrographs acquired after dynamic monitoring. Despite the presence of tortuous vessels characteristic of tumor tissue (a), a nonnegligible amount of areas in the tumor tissue did not show any doxorubicin signal (b). Figure S6: representative fluorescence micrographs (mosaicking) of a tumor tissue exposed to TSL. DOX distribution is heterogeneous. Objective: 10x. [file 2645928.f1.pdf]

Supplementary materials

Rat number	DOX administration	Fluorescence during monitoring	Fluorescence after monitoring	DOX level in urine (μg/mL)	DOX level in blood (μg/mL)
1	free DOX	none	none	2.9	not detected
2	free DOX	none	none	not detected	1.3
3	free DOX	none	none	1.9	not detected
4	TSL	yes	yes	4.3	15.1
5	TSL	yes	yes	3.4	3.1
6	TSL	none	yes	not collected	11.2
7	TSL	none	yes	not collected	1.7
8	TSL	none	none	2.4	not detected

Table S1: Summary of the results rat-by-rat. DOX concentrations in urine and blood correspond to the results of single quantitative measurements.

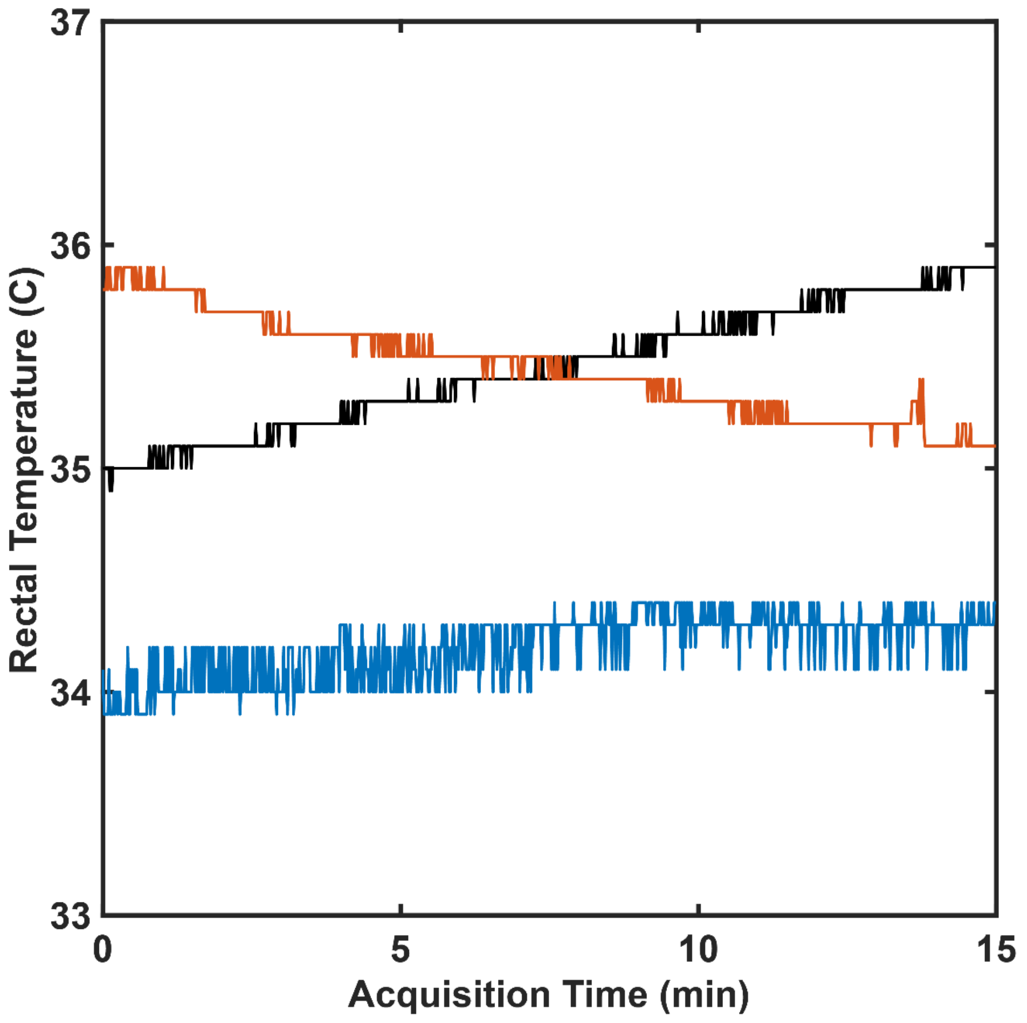

Figure S1: Representative profiles of rat rectal temperature recorded during real-time monitoring.

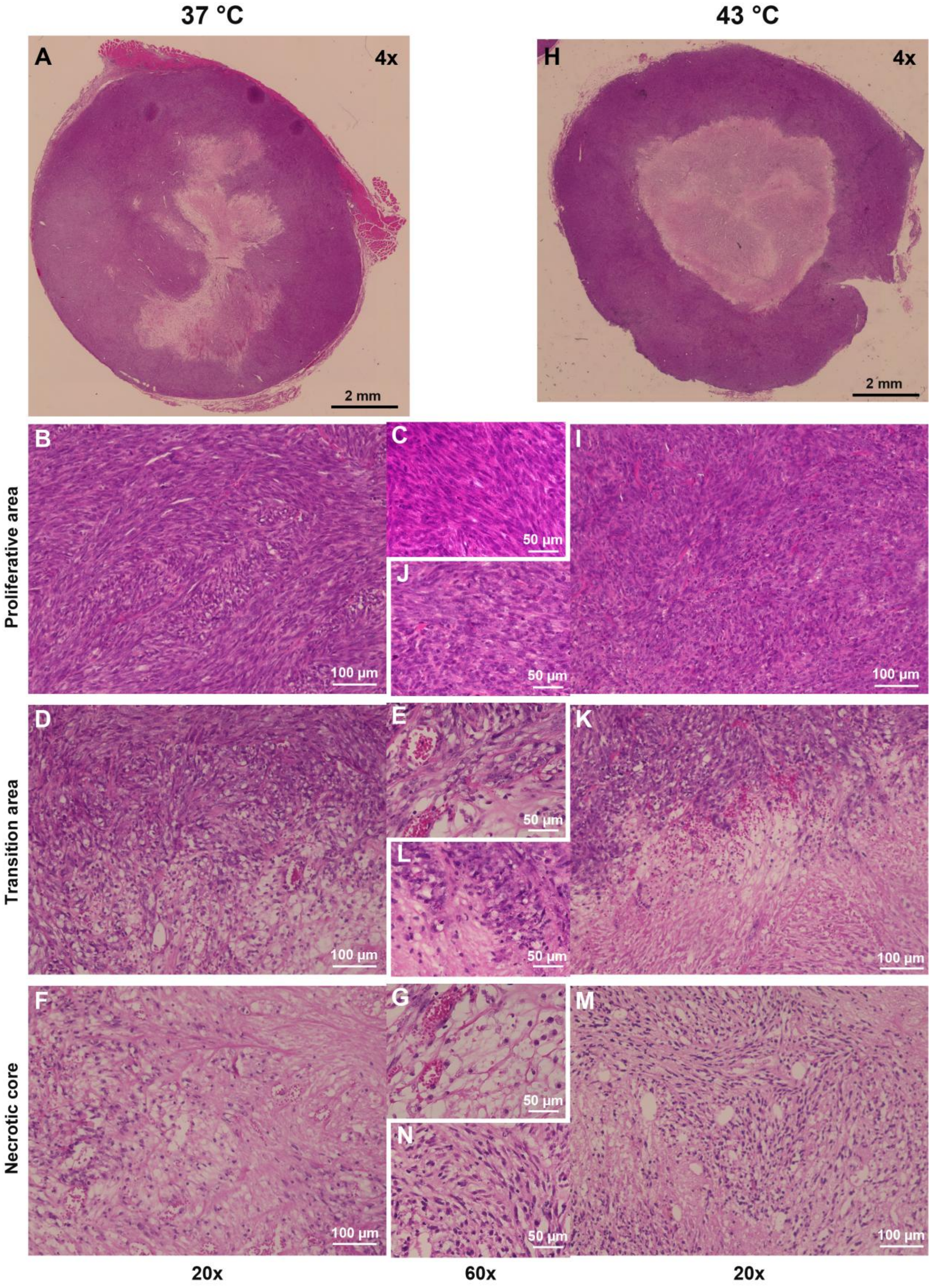

Figure S2: Micrographs showing the impact of mild hyperthermia on the tumor microstructure in the proliferative area (b,c,i,j), the transition area (d,e,k,l) and the necrotic area (f,g,m,n) of the rat 4. No morphological damage was observed at 43 °C (h-n) compared to 37 C (a-g) in any of the 3 areas.

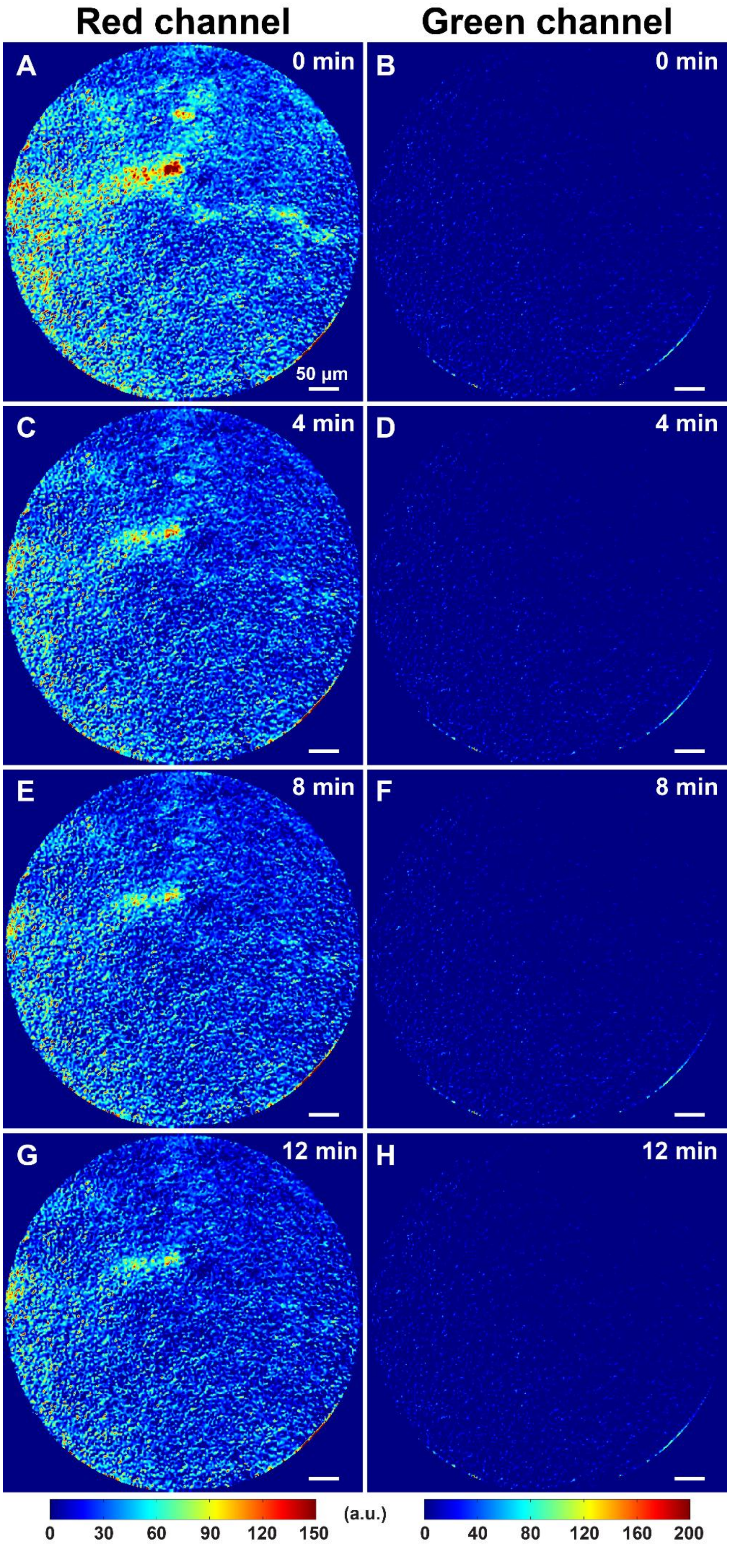
Red channel
Green channel
A
0 min
B
0 min
50 µm
C
4 min
D
4 min
E
8 min
F
8 min
G
12 min
H
12 min
0 30 60 90 120 150
(a.u.)
0 40 80 120 160 200

Figure S3. Real-time monitoring of doxorubicin penetration in the tissue microenvironment after free DOX intravenous injection. a,c,e,g). Red channel with AngioSense^{TM} (blood-pool labeling) showed that the acquisition was performed in a steady FOV). b,d,f,h) No DOX fluorescence signal enhancement could be observed in the green channel after free DOX intravenous injection.

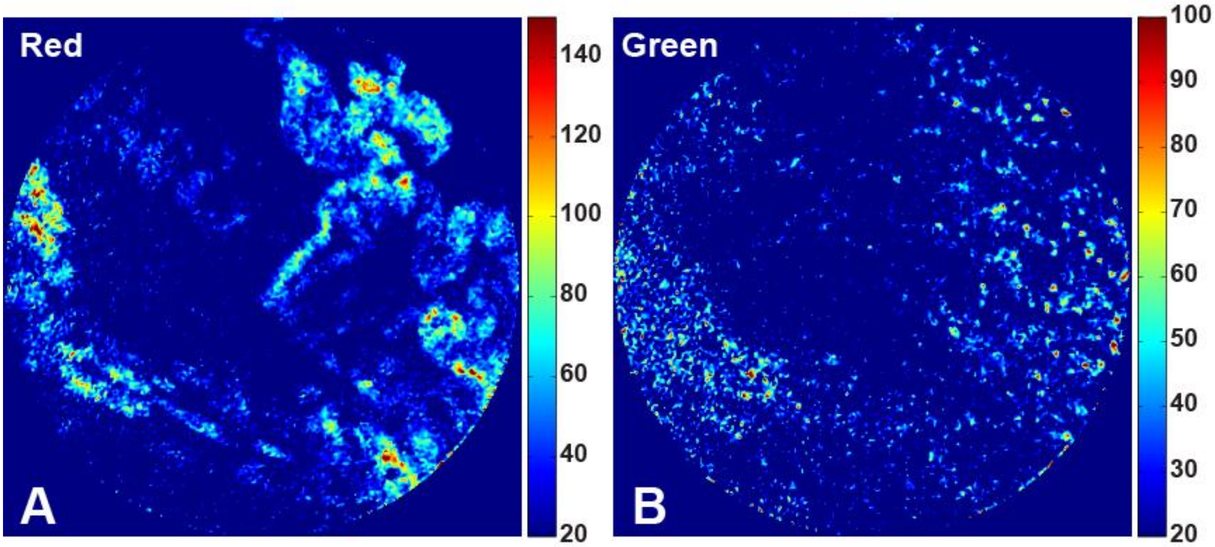

Figure S4: Micrographs collected after dynamic monitoring. In the interstitium, nuclei that take up doxorubicin (b) are predominantly located around the vessels (a).

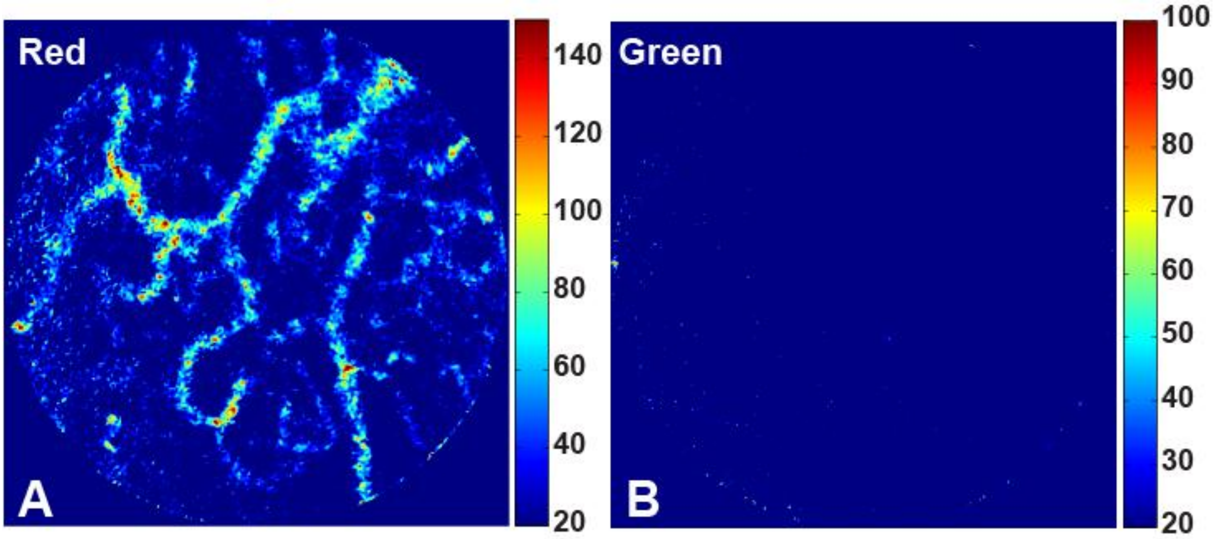

Figure S5: Micrographs acquired after dynamic monitoring. Despite the presence of tortuous vessels characteristic of tumor tissue (a), a non-negligible amount of areas in the tumor tissue did not show any doxorubicin signal (b).

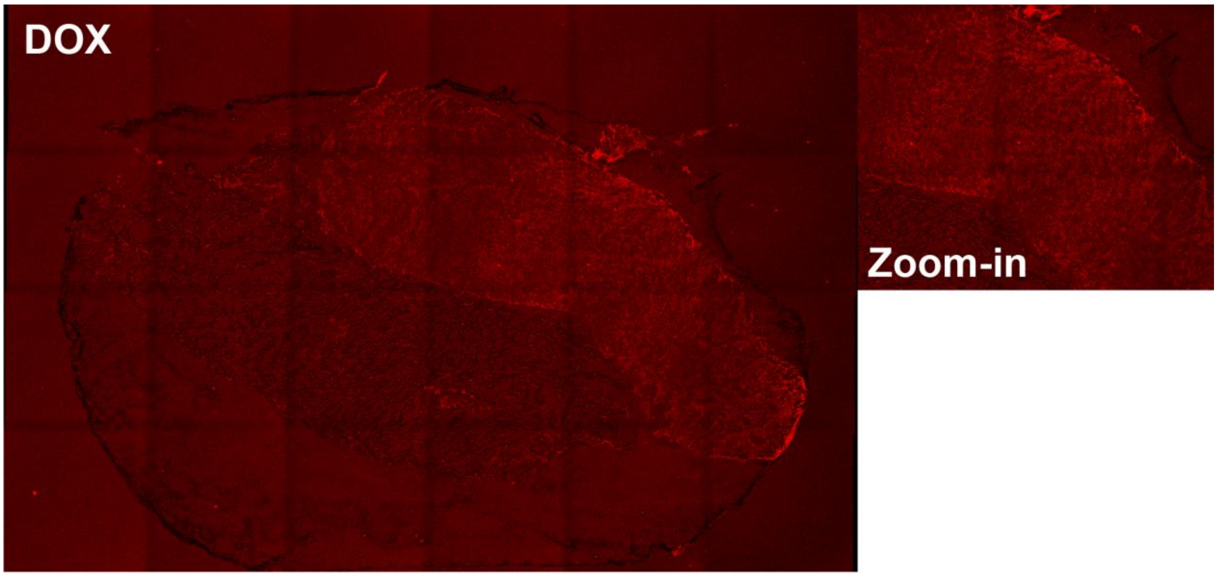

Figure S6: Representative fluorescence micrographs (mosaicing) of a tumor tissue exposed to TSL. DOX distribution is heterogeneous. Objective: 10x.
